# Experiences and Psychosocial Impact of West Africa Ebola Deployment on US Health Care Volunteers

**DOI:** 10.1371/currents.outbreaks.c7afaae124e35d2da39ee7e07291b6b5

**Published:** 2016-09-21

**Authors:** Robyn Gershon, Liza A. Dernehl, Ezinne Nwankwo, Qi Zhi, Kristine Qureshi

**Affiliations:** Philip R. Lee Institute for Health Policy Studies and Department of Epidemiology and Biostatistics, School of Medicine, University of California, San Francisco, California, USA; Department of Global Health Sciences, Graduate Division, University of California, San Francisco, San Francisco, California, USA; Philip R. Lee Institute for Health Policy Studies and Department of Epidemiology and Biostatistics, School of Medicine, University of California, San Francisco, California, USA; Philip R. Lee Institute for Health Policy Studies, School of Medicine, University of California, San Francisco, California, USA; Global Health Nursing, School of Nursing and Dental Hygiene, University of Hawaii at Manoa, Honolulu, USA

**Keywords:** ebola, health care workers, infectious disease, mental health, preparedness, volunteer, West Africa

## Abstract

Background: This qualitative study was designed to assess health care volunteers’ experiences and psychosocial impacts associated with deployment to the West Africa Ebola epidemic.

Methods: In 2015, using snowball sampling, 16 US health care volunteers who had recently returned from West Africa were recruited for this study. Semi-structured interviews were conducted to collect information associated with each phase of deployment (pre, peri, and post).

Results: Participants reported that they were motivated to volunteer because of a sense of responsibility and feelings of empathy and altruism. Immediately prior to deployment, most reported fear of contagion and death, as well as doubts regarding the adequacy of their training. Family members and close friends expressed high levels of concern regarding participants’ decisions to volunteer. During the deployment, participants were fearful of exposure and reported feeling emotionally and physically exhausted. They also reported feeling frustrated by extreme resource limitations, poor management of the mission, lack of clearly defined roles and responsibilities, and inability to provide high quality care. Upon return home, participants felt a sense of isolation, depression, stigmatization, interpersonal difficulties, and extreme stress.

Conclusion: Preparedness of volunteers was suboptimal at each stage of deployment. All stakeholders, including volunteers, sponsoring organizations, government agencies, and professional organizations have a shared responsibility in ensuring that volunteers to medical missions are adequately prepared. This is especially critical for high risk deployments. Effective policies and practices need to be developed and implemented in order to protect the health and well-being of health care volunteers to the fullest extent possible.

## INTRODUCTION

Infectious disease outbreaks, epidemics and pandemics are increasing in frequency and severity.^1^^,^ 2 ^,^ 3 Many, if not most, of these bioevents have been caused by ‘emerging pathogens,’ which are described as newly appearing or reappearing in the population, or rapidly increasing in incidence or geographic range.^4^^,^ 5 In the past few decades alone, more than 85 emerging pathogens have been identified, including HIV, multidrug resistant tuberculosis (MDR-TB), West Nile Virus, Hanta virus, severe acute respiratory syndrome (SARS), H1NI influenza, Middle Eastern Respiratory Syndrome (MERS), Chikungunya virus, and, most recently, Ebola and Zika viruses.^3^^,^ 6 ^,^ 7 ^,^ 8 The risk factors for the increasing incidence of bioevents worldwide include human population growth, inadequate health care and public health infrastructure, increased globalization, land-use that facilitates zoonotic transmission, climate change, declining vaccination rates, and overuse of antibiotics.^9^

Potentially lethal bioevents present significant risk of morbidity and mortality to exposed populations and to health care workers who may come into direct contact with infected patients or their specimens. The risk of exposure and infection is especially of concern in the early stages of outbreaks, as information regarding transmissibility may be limited, while, at the same time, effective vaccines or other pharmaceuticals may not yet be available.

When bioevents occur in countries with limited health care resources, the international community is generally highly responsive in providing much needed support to these ‘hot zones.’ This type of assistance was especially important during the 2014-2015 West Africa Ebola epidemic. Within a week of the initial case report in April 2014, the Centers for Disease Control and Prevention (CDC) sent an expert team to Guinea. Within the following months, over 2,000 other CDC employees deployed in response to outbreaks in Guinea, Liberia, and Sierra Leone, with many more working on the public health response from the CDC headquarters in Atlanta, Georgia.^10^ Other governmental agencies (e.g., United States Agency for International Development) from the United States (US) and many other countries, and more than 65 non-governmental agencies, most prominently, Médecins Sans Frontières (MSF)/ Doctors Without Borders and Partners In Health, also sent health care volunteers on medical missions to West Africa to respond to the epidemic and to provide care to patients with Ebola virus disease (EVD).^11^ It is estimated that ultimately thousands of health care workers from throughout the world voluntarily deployed to the West Africa Ebola epidemic.

Even though the health care response was substantial, there was some concern within the international public health community that a more rapid and robust response was warranted.^12^ Local West African health care systems were quickly overwhelmed, and the result was deadly. As of May, 2016, over 11,000 deaths were reported among the estimated 28,000 suspected, probable or confirmed cases of Ebola, (average case fatality rate =39%).^13^ According to a 2015 World Health Organization (WHO) report, infected health care providers had an even greater risk of mortality; during the epidemic, over 50% (418/814) of Ebola infected health care workers died, with the highest case fatality rates (68%) observed among nurses (193/285).^14^ The impact on the health care workforce in affected countries was sizeable. According to a Lancet report published in July 2015, Guinea lost 1.45% of their healthcare workforce, while Liberia and Sierra Leone lost 8.07% and 6.85%, respectively.^15^ It is important to note that in some of the areas severely affected by the Ebola epidemic, health care access was already severely limited, with roughly only one or two doctors per 100,000 people.^16^

The US health care response to the Ebola epidemic exemplifies the country’s long tradition of humanitarian health care volunteerism. Although there is no mechanism currently in place to track this, an estimated 200,000 health care personnel from the US are believed to volunteer each year to underserved or disaster-affected areas around the world.^17^ Many volunteer through the more than 1000 non-governmental sponsor organizations, which may be faith-based, university-based, or sponsored by educational or corporate entities. These organizations recruit through their internet presence, word-of-mouth, or through targeted advertising. A recent survey of 177 voluntary health care organizations found that more than half accepted 75% or more of all health care applicants into their volunteer programs.^17^ The same survey also found a wide range of preparation provided to volunteers, ranging from simply providing an ‘orientation packet’ of materials to extensive, in-person training. For Ebola deployments, the CDC developed an intensive experiential training course for US health care workers deploying to West Africa; over 600 US workers eventually received this training.^18^ Similar courses were developed by MSF and WHO.^19^^,^ 20 Even with this extensive training in place, some experts, including leadership at numerous US academic medical centers, urged caution to staff and fellows considering voluntary deployment. This was due to concerns regarding the effectiveness and implementation of safety precautions, the high fatality rate, and the lack of effective treatments at that time.^21^

In general, most US humanitarian health volunteers report very positive deployment experiences. Anecdotally, some US volunteers to the West Africa Ebola epidemic similarly had positive experiences and reported feeling ‘privileged’ and ‘grateful’ for having had the opportunity to serve.^22^^,^ 23 ^,^ 24 ^,^ 25 However, research on the experiences and especially the emotional impact of deployment to the Ebola epidemic among US health care professionals is very limited. Adverse psychosocial impact of deployment on disaster volunteers has been well documented, even among highly trained and well prepared volunteers.^26^^,^ 27 This study was designed to improve our understanding of the impact of health care deployment to high risk outbreaks. Strategies suggested here for reducing risk of adverse effects in volunteers may be applicable to other deployments to international ‘hot zone’ disasters.

## METHODS

*Study design*

Semi-structured interviews were conducted with US health care workers who had recently returned from voluntary deployment to the West Africa EBV outbreak. Data were analyzed using qualitative techniques.

*Study participants*

Potential participants were identified using a snowball sampling strategy and contacted via email. In this initial contact, the purpose of the study was explained, as well as the study methods, the human subjects protections in place, and the eligibility criteria for participation, which included recent deployment to West Africa Ebola epidemic [Liberia, Sierra Leone, and/or Guinea], and current employment as a health care worker in the US. This study had prior approval from the Office of the Committee on Human Research at UCSF (study # 15-15914). Participant characteristics are shown in Table 1.

**Table 1:** Table 1. Characteristics of the sample (N=16)

Characteristic	n (%)*
Role	
Nurse	5 (31)
Physician	11 (69)
Gender	
Male	9 (56)
Female	7 (44)
Age	
20-29	1 (7)
30-39	6 (40)
40-49	3 (20)
50-59	2 (13)
60-69	2 (13)
>70	1 (7)
Deployment Country	
Liberia	9 (56)
Sierra Leone	7 (44)
Guinea	1**
*Valid %	
**One participant was deployed to two of the affected countries	

*Data collection*

Drawing upon available information on humanitarian health care, a semi-structured interview guide was developed. Sixteen interviews lasted an average of 45 minutes in length, and were conducted from May to July, 2015, via phone, Skype, or in-person. All interviews were audiotaped with prior knowledge and approval of the interviewee. Participants were informed that the tapes would be destroyed upon completion of the study. Each participant was assigned a unique code number, and no personal identifiers of any kind were noted in the tapes or transcriptions. All study materials were stored in password-protected files on an encrypted computer only available to the research team. All study materials are available by contacting the corresponding author.

*Data analysis*

Qualitative thematic analysis was used as the analytic framework for identifying themes relevant to the experiences of the participants before (pre), during (peri) and after (post) their deployment. Two research team members reviewed the first three transcripts to develop a list of codes and a coding manual that defined the common terminology to describe the themes identified. The remaining transcripts were then inductively open-coded by two independent reviewers, followed by thematic analysis using the ‘constant comparison’ methodology.^28^ To help ensure that the coding was both valid (i.e., well grounded in the data and supportable) and reliable (consistent in meaning), the criteria for assigning a specific code to a block of text was well documented. The coding scheme (and codebook) was refined and expanded upon to reflect and incorporate emerging insights throughout the coding process. Discrepancies in coding were discussed and mutually resolved.

## RESULTS

Pre-deployment

*Motivation to volunteer*

The most frequently cited motivation for volunteering was the belief that the participant’s skills were needed and therefore there was a perceived ethical obligation to volunteer. As noted by one participant, “For me, it was just the right thing to do. I felt like I had something that I could do to help and I wanted to do it.”

Several participants noted a commitment to social justice and health equity and were thus drawn to serve people in need. Experiences with other humanitarian health crises, usually in the aftermath of disasters, such as the 2010 Haiti earthquake, or past experience on medical missions in other parts of Africa, were also motivators. One participant stated, “Having worked there previously, having had very close West African doctor friends die… you can’t just stand there and bear witness.”

*Fear for self and fear and concern of family and friends*

Even though participants were highly motivated to volunteer, early unfolding reports^29^^,^ 30 led many participants to question their decision to volunteer. Instead of backing out, several said that they just resigned themselves to going even though they had a sense of foreboding and fatalism about the deployment; “The week that I was going to go, the CDC said 1.4 million people could be infected. I saw that and thought, “I’m not coming out of this….”

Family members, especially parents, did not want them to go. This led some participants to avoid telling anyone about their decision. In some cases, secrecy was extreme, as recounted by one participant, “I didn’t tell my parents…. until I was in the airport leaving.” Another participant similarly did not want to tell people about her upcoming deployment, “I didn’t really talk to anyone about it when I left. I had a very morbid feeling, you know, I was so scared…I don’t know, it felt too morbid and that it would be like …. oh we better say goodbye in case she doesn’t come back.” Instead of reassurances from family and friends, negative reactions like these were common, as one participant recalled, “There was so much hysteria….people thought I was going to die. So it felt like you were jumping off a cliff.”

*Training*

The length and quality of training varied. In the very early stages of the epidemic, participants already working in other parts of Africa reported that they received their training in other (non-affected) countries, typically provided by their sponsoring agencies. Others received training at CDC with additional hands-on training at the deployment site. Some participants (especially those who had deployed early in the epidemic) felt that they had not received adequate training, and thus felt unprepared. As reported by one participant, “They (the sponsoring agency) handed me a viral hemorrhagic fever guide. I read it on the plane, showed up, but I had no real idea of what I was doing.”

As the outbreak progressed, CDC began offering extensive training at a facility in Anniston, Alabama, and all of the subjects who took this training spoke very highly of it, especially with respect to their confidence in using the required personal protective equipment (PPE). Training on the complex process of donning and doffing PPE was universally regarded as critical in keeping them safe, as was the simulated practice of working while wearing the PPE.

Several study participants thought that their training did not address their perceived lack of cultural competency. As reported by one participant, “There’s a lot beyond the training [on infection control practices] that I felt completely unprepared for… there could have definitely been a lot more preparation on a cultural level…in terms of cultural norms and acceptances, language, and perceptions of westerners…. that would have been extremely helpful.”

Peri-deployment

*First impressions*

By any measure and at multiple levels, the early humanitarian response to the Ebola epidemic was extraordinarily challenging. Health care facilities and systems, already severely under- resourced in the affected areas, were strained to the limit. US health care workers, even those with prior humanitarian deployments, were shocked by the local conditions. One participant, who deployed very early in the epidemic (August, 2014), described his first impression of the Ebola Treatment Unit (ETU) where he was assigned, as “a real horror show.”

Living conditions were unexpectedly austere, even for those who had previously volunteered in other resource-limited countries. Most participants said they had been provided with a bed and a mosquito net, but that running water, potable water, electricity, working toilets, and showers were scarce, making it difficult to maintain personal hygiene.

*Lack of organization and role clarity*

Lack of organizational management of the medical mission was reported. Some noted that the people in charge were making poor decisions, especially regarding scheduling and assignments. These problems were compounded by a lack of communication. Clear policies and procedures were lacking; as one participant noted, “It seemed like policies were being formed by whoever was in charge. The person in charge changed every three hours.” Because of severe staff shortages, participants had to perform new types of tasks. One physician noted, “I hadn’t put in an IV in probably 10 years. I had to mix all of the drugs myself. That is not typically part of my daily workload. That was certainly stressful.” Others reported feeling underutilized, one nurse stated, “I felt like I wasn’t a very effectively used resource in the sense that I spent like 2 weeks of my time there doing kind of menial tasks because they didn’t have another role for us to play.”

*Lack of resources*

Before international teams arrived to help set up functioning ETUs, the delivery of care was extremely constrained not only by a lack of staff, but also by a lack of basic resources (medical supplies and equipment), infrastructure (facilities lacking beds, running water, sanitation, electricity, etc.), and systems management (protocols for care delivery). As one participant noted, “it was an absolutely resource limited setting.” In some ETUs, patients were not housed based on their health status but by the availability of beds (or, in some cases, cots or mattresses on the floor). Family members were placed in the same room, regardless of their health status. As recalled one participant, “Seeing the horror of a person who had Ebola, yet still alert, sharing the same room as family members succumbing to EVD, was just a terrible, terrible thing to see.” Because it was not feasible to have health care workers continuously monitoring the patients housed in the ETUs, sometimes patients were in the same room for extended periods with others who had died from Ebola. Many patient deaths, including deaths unrelated to EVD, were thought to be directly linked to a lack of resources, as retold by one participant;

“I remember one baby who was about four months old, suspected of EVD… as the mother had died of it. At that time, because we were minimal staff, and because of the lack of electricity and concerns for safety, we did not have a night shift. This baby was lying on a mattress by itself, obviously really dehydrated and soiled. We cleaned the baby, tried to feed the baby, …and we didn’t have any diapers, any baby bottles, we didn’t have small enough IVs to even attempt an IV…We went in several more times that day, fed the baby with a syringe, gave it some acetaminophen… The next morning [we] went in and the baby was dead. In my mind, the baby basically starved to death because the baby wasn’t symptomatic for Ebola.”

*Feelings of depersonalization*

These types of extreme experiences led some participants to feel emotionally withdrawn from their patients. One participant noted, “I felt like this was the only way to protect myself and continue to be productive. It’s really hard to desensitize yourself like that, but you have to…. as a protective measure.”

*Fear of contagion and illnesses suggestive of EVD*

Thoughts of getting infected were the uppermost concern for most, especially during the beginning of the deployment when they were still becoming acclimated to the ETU and whenever there was a breach in infection control protocol and practice. For some, fear was constant. One participant recalled constantly thinking, “Don’t let me get Ebola, don’t let me get Ebola.”

Many reported getting sick during their deployment, with symptoms that were suggestive of Ebola infection. Fortunately, none of the participants in this study became infected with Ebola (there have been nine cases of EVD reported among US health care volunteers, with one death).^31^^,^ 32 Most of the illnesses experienced during deployment were believed to be related to contaminated food and/or water.

*Difficulties with the personal protective equipment and infection control protocols*

The donning and doffing procedures for PPE were time consuming, taking about 20-25 minutes before and after working in the ETU. The PPE was also reported to be uncomfortable and claustrophobic. In spite of the limitations of the PPE, strict adherence to infection control measures within the ETUs was strongly encouraged, as one participant noted, “[When] you’re getting ready to go in, all the nurses would do a huddle and make a plan, decide who was going to do what…and then we would say a prayer as a group before going in…basically for a safe return and see you on the other side.” Several noted that by the time they actually entered the ETU, they were already sweating profusely and their goggles had fogged up. High heat and humidity made it impossible for most to work in the ETUs any longer than 2-3 hours. The PPE was also seen as a barrier to patient care. One participant summarized her experience with PPE, “You do the best that you can with body language and voice…you’re always in a hurry. After an hour, you were really putting yourself at risk for passing out because of the heat. Always being rushed by that clock ticking… and I think we all felt guilty about rushing out, but there was just no way around it.”

*Altered standards of care and ethical conflict*

Many of the participants remarked that the hardest part of delivering patient care in the ETUs was the adjustment to altered standards of care. In the US, we adhere to basic and fundamental ethical principles of patient care, which include respect for persons and their autonomy, the concepts of beneficence, non-maleficence (do no harm), justice and fairness to all patients. While we recognize that emergency situations may require alterations to usual practices due to resource limitations, many of the participants reported that nothing could have prepared them for the constraints that they now faced. The inability to provide the quality and level of care that participants were used to delivering was an important source of stress and frustration. As noted by one participant, “Even IVs were considered to be a very invasive procedure…there was a ward where people could go to get them…not very many people got to go to that ward—even if they were critically ill. They still had to be healthy enough that we could justify taking them to the IV ward…. they didn’t qualify for IV because they’re probably going to die anyway.” Nevertheless, some participants manage to adapt. One nurse reported that she was able to keep going by constantly reminding herself, “I’m doing the best that I can with what I have, and that’s all I can do.”

Witnessing unnecessary patient suffering due to shortages of drugs and other resources was extremely disturbing to all participants. Lack of pain medications was especially concerning, as reported by one participant, “We didn’t have any narcotics, so pain control was really challenging. We didn’t have fentanol, we didn’t have morphine. We didn’t have anything like that.” Others recalled wishing they could give their patients any small measure of comfort, as one nurse said, “Even little things could mean a lot. Like to get a cold Coke would be an amazing treat. But we didn’t have these little things that would have made them feel so happy and maybe even help them a little bit. That was hard.”

Another key source of concern for many participants was witnessing undignified death. As noted one participant, “It’s one thing to die alone, and then it’s another thing to die in an environment where its hot and dark, and theres plastic tarps and chlorine smell, and you’re on a cot as people come in wearing these big suits and leaving. And to die that way after suffering for days by yourself… I remember that very vividly.”

While all the participants had prior experience with patients dying, none had previously experienced mortality rates this high. As one participant recalled, “Literally the bodies were stacking up outside the ward…I mean... there were many days when there were six to eight occupied body bags lying outside the ward… We just had too many bodies.” One physician recalled that, “50% of the patients on the [60-90 patient] ETU died...… every day.”

*Safety and security*

In our sample of 16 participants, over 80% reported a breach in PPE while working in an ETU. Although the ETU was the most common place that subjects felt at risk of exposure, there was also the potential for exposure out in the community. As one participant recalled upon reporting to a local community health center, “I walked in on a local doctor caring for a patient who clearly had Ebola. He had decided to treat the patient there because there were no beds available in the ETU. I didn’t touch anything, but still, I walked in. I could imagine all kinds of ways that I would have had no idea that Ebola was right there under my nose.”

The prevention of Ebola exposure, while a common cause of concern for participants, was not the only concern. Maintaining personal security was also a source of worry. One participant was the victim of a violent mugging: “I got jumped and mugged and had everything stolen...passport, credit cards, everything...… there were some bruises and what not, but it was more mentally what it did to me. I actually thought I was going to die. I couldn’t trust anyone anymore.”

Several participants mentioned feeling threatened by community members who were angry and afraid of the ETUs and the health care personnel working there. Family members of ETU patients also sometimes threatened violence. Driving from one location to another was an additional cause for concern. As recalled one participant, “There was definitely a sense of hostility in some of the deployment areas. Even though we had the Ebola Team sign on the car, there were some checkpoints where we had to get out of the car and hand over our passports. There was anxiety about what would happen.”

*Fatigue*

The workload was very taxing, as staffing was limited and patient load extremely heavy. At least two participants said they started their deployment working 12 hour shifts, six days a week. One participant reported that in his ETU, which had over 70 patients each day (even though it was originally designed for 14 people) - there were only two staff members. Because of the staff shortages, participants said they felt guilty if they took a day off to rest, as noted by one participant, “The one day off a week policy was there, but it was on an honor system that many didn’t abide by. Because it’s the guilt you feel… obviously the local health care workers aren’t taking that day off, patients aren’t taking that day off so…it was initially recommended, and eventually required, as the strain became more apparent.” There were a couple of times where participants felt so overwhelmed and exhausted that they had considered leaving; as one noted, “we had discussions about whether or not we should stay, in the end we always decided to stay.”

Post-deployment

*Quarantine and sense of isolation*

Most participants reported that their reentry experiences were very stressful, as illustrated by the following recollection: “The Airport was just awful… they sent you back into this little office … I coughed one time. That led to me being put into a room that was the equivalent of a jail cell: metal bench, metal toilet without a lid, and a camera. Two doctors in full PPE came in and questioned me.”

In our sample, once participants returned home, they were placed in home quarantine. This was also reported as an extremely stressful time. As one participant noted, “I had to do a 21-day mandatory quarantine and observation period. I actually, oddly, found this to be the most traumatic part of the whole thing. I come from a very small town and everyone kind of knows everyone else’s business. My family was basically convinced that I was going to die of Ebola at any moment. I did my quarantine at my parent’s, but I had to have my own room, my own utensils and plates, my own bathroom, I wasn’t allowed to touch anyone. I had to maintain a three foot radius at all times. I wasn’t even able to have a very close conversation.” Another participant mentioned that she lost a lot of friends during quarantine, because, “nobody would even pick up the phone... It was just awful.”

Lack of physical contact during the quarantine was problematic for some. Many had not had any physical contact for many months. One participant reported that she had not touched a single person from early November until the end of January, “And then suddenly… 21 days was over. My public health nurse came to my house on day 21 and we realized it was over….and I don’t have Ebola and she gives me a hug… and I was like…oh my God…you’re the first person that has touched me in 3 months and now…..like I’m terrified. I miss my 3 foot bubble just because I felt very secure. Now when someone reaches out for a handshake or a hug, … I’m like, are you sure you know what you’re doing?”

Even after the quarantine period was over, some wanted to be alone, as recalled one participant, “As soon as the quarantine was over, everyone wanted things to go back to normal, as if nothing had happened….[but], I didn’t want to see anyone. I was so used to being alone.” Still, at least one participant reported more positive experiences upon their return, “most people were great, the problem was the 5% who were freaks about it.”

*Stigmatization, lack of appreciation*

Participants reported that they felt ‘anger’ and ‘resentment’ from their family members and friends upon their return. They felt unprepared for the reactions of those around them, as expressed by one participant, “No one wants to be around you- even after the quarantine is over. I was like, oh my gosh, people don’t want to be around me. I felt very naïve and stupid for not foreseeing it. Like I was making plans like nothing was wrong, but nobody wants to deal with you.”

Some wanted validation of the risks they had taken by volunteering. They wanted the public to know what they had been through and how they had put their own lives at risk to help protect others. Participants felt that their efforts not only helped people in West Africa who were directly affected, but that they also helped to prevent the spread of Ebola to other countries, including the US. They wanted the authorities to confirm that the volunteers were not reckless, but rather, that they were selfless. They felt like heroes, and wanted to be appreciated for their volunteer efforts, but instead, some reported feeling stigmatized upon their return because, as one participant stated, “People were being selfish, and only worried about their own health, and not how the Ebola response effort was about the health and well-being of total populations.” Another participant felt that the attitude in the US was, “I’m in the US and this doesn’t involve me, so why should I care?”

Several participants experienced stigmatization, as did their family members. As reported by one participant, “Not only was there fear and stigmatization against me, but against my family as well. My mom volunteers at a school…. they told her not to come in.”

*Grief, depression and guilt about leaving*

Participants reported feelings of grief, mourning, sadness, depression, remorse, and regret upon their return. As one participant said, “Oh, we could have done much, much more.” Others reported feeling as if they were on an emotional roller coaster, as one participant noted, “I felt disappointed and defeated. Other times, I felt, ‘Ok, I did everything I could, this is good’. So up and down. It was a nightmare.” Another participant described how he had felt upon return, “I was a total mess because it was right back to work. Everyone wanted to tell me how amazing I was, but I felt just horrible inside. It was hard to dialogue with people about it.” Difficulty sleeping was also reported to be a problem for several participants once they returned home.

Several mentioned that they had been offered mental health services upon return, although most did not utilize it. The reasons for not using varied, from “I didn’t think I needed it at the time,” to “it was too impersonal, by phone, by skype.” Those that did use the offered services found them to be very useful. One participant was particularly grateful to an occupational medicine physician who called and checked on her well-being for days and weeks after she returned home. But, in general, most participants did not feel that they had received adequate counseling resources.

Some expressed remorse about leaving their Ebola deployment when their assignment was completed. As noted one participant, “I did not want to leave. After 9 weeks there, it was really hard to leave. There were still so many people needing care, still so many cases and so much to do. I felt like I was abandoning the people there, including the nationals still working there.”

*Self-doubt, disillusionment, re-entry stress, symptoms of PTSD, reflections*

Upon return, several participants started doubting the meaningfulness of their deployment. One participant reported, “I had this whole questioning of myself and my role in humanitarian response…and then just the stigmatization following all of it…. You know I kind of came to the fact that all of this is dirty, and humanitarian response is going to have some of its dirtiness. Nothing is as altruistic, clean and fair as you think it is. Everything is complicated and not everybody is in it for the purpose of helping each other out.” Another participant noted, “[the deployment] really was an eye opener. It showed me where the deficits are within all these international [humanitarian] organizations.” Many were dismayed by the inequalities they saw between the US health care workers and local health care workers, especially in terms of treatment when a worker became ill. They also were upset by the fact that local health care workers sometimes did not receive their promised pay.

Transitioning back to everyday lives was difficult for many. As one participant recalled, “When you come back, you don’t have that grieving time. You don’t work through any of the emotions and everything. You just put everything on hold.” At that time, there just wasn’t any time for debriefing- to help reacclimatize returning to your homes. In hindsight, I think it would have really been good to have had debriefing.” Others mentioned feeling isolated because the only people that they felt they could really talk to and who understood what they were feeling were the people who had deployed with them. As noted by one participant, “You breathe, you eat, you sleep it, for 24 hours of every day. It’s not like you can come back home and relax with your family. Your heart is just not into it.” Another participant similarly found his reentry disquieting, “Reentry was really really rough. I spent a lot of time just sitting in my office. Staring out of the office. I mostly internalized it. I drank a lot.”

Several doubted they would do another deployment like this, and if they did, certain conditions would have to be in place, as one said, “I’d have to know that what I was doing was making such a huge difference that it was worth it to be freaking miserable for 21 days.” Sadly, others reported a loss of sense of purpose in life once they returned, as one physician reported, “Simply put, I’ve just lost my way. When I got back, the problems were still there, but it felt like I was just finished with the way I had been living. No worries—you’re gonna die anyway in this outbreak. Reforming a new life has been tough. I guess you could call it PTSD. I’m proud of what I did…. but in my personal life, I’ve paid a heavy price.”

*Positive reflections*

Despite these predominantly negative findings, some positive experiences and feelings were noted as well. For instance, almost all participants reported a very strong sense of comradery and teamwork within their group. There were also many reports of feeling a great deal of respect, admiration and appreciation for the skills, commitment and hard work of the nationals and other team members that they worked with. In spite of the personal toll of the deployment, several said were inspired to continue to volunteer, as reported by one participant, “It renewed my commitment to health justice, and health equity for everyone,” and, as noted by another, “I would volunteer again, now that I have a better understanding of what needs to be done in an outbreak both in terms of preparedness and response.”

## DISCUSSION

In this study, we found important gaps in preparedness at each stage of deployment, with perhaps the most important deficiencies noted in the pre-deployment phase. One of the difficulties in making recommendations for improvement is determining who the recommendations should target. Is it the sponsoring agency? Is it the person wanting to volunteer? Is it the professional organization representing these US health care workers? It seems reasonable to assume that the responsibility for ensuring a safe and effective high risk deployment should be shared among all key stakeholders.

Most importantly, the international community, specifically, WHO has a responsibility for ensuring efficient and rapid response to future outbreaks. In particular, the WHO Department of Pandemic and Epidemic Diseases provides strategic leadership in support of affected countries with limited resources. The WHO, working in concert with their international partners, should develop preparedness recommendations that aim to protect the health and well-being of all health care volunteers. Recommendations should target the pre-deployment stage, starting with the provision of clear, accurate, and realistic information about the mission, the conditions in the field, and the steps that will be taken to help protect the health and safety of volunteers.

Sponsors of medical missions are responsible for providing high quality training, such as the training developed and provided by the CDC and other organizations in response to Ebola. Hands-on simulated training is essential for complex infection control practices that may be unfamiliar to most health care volunteers. This training should be mandatory and verified (preferably by high level US public health agencies) prior to any high risk deployment. Specialized counseling at the pre-deployment stage should be targeted towards both volunteers and their families in order to help reduce fear, anxiety, and resentment of family members. Pre-deployment counseling might also help volunteers cope with other types of stressors they may commonly face during the deployment (fear, work-related problems, mental and physical exhaustion), as well as after the deployment (quarantine challenges, shunning, stigmatization, and sense of isolation). Volunteers must be provided with full disclosure of the procedures that will have to be followed upon return in terms of quarantine. This is especially important because the US does not have extensive experience or uniformity with regards to quarantine policies or procedures, and there was (and remains) a lack of standardized management of health care volunteers returning from highly contagious and lethal infectious disease deployments.^33^^,^ 34 Volunteers to hot zone missions need to know what to expect upon initial reentry into the US, and upon return to their particular home state and local jurisdiction, as quarantine laws vary considerably around the country.^35^ For large scale events, the CDC has an important role to play to help reduce stigmatization of returning volunteers and their families by launching general public information campaigns. These types of campaigns might better prepare communities to both send and receive returning volunteers. Sponsoring organizations also have a responsibility in assuring that there are support systems in place for volunteers (and their families, if needed) upon their repatriation back home.

Health care workers interested in volunteer work similarly have important preparedness responsibilities. First, it may be prudent to consider volunteering locally or nationally before agreeing to overseas missions. Because the US is one of the top five nations worldwide^36^ in terms of natural disaster occurrences, there are many opportunities for health care workers interested in volunteering for disaster response. For example, the National Disaster Response System (NDMS), which include the Disaster Medical Assistance Team and the National Nurse Response Team are excellent volunteer options to explore.^36^ Another good option is the Medical Reserve Corps (MRC),^37^ a national network of volunteers that are organized locally to improve the health and safety of their communities. Health professionals can also volunteer through the American Red Cross.^38^

Before considering an overseas medical mission, potential volunteers should review as much information provided by the sponsoring organization as possible, as well as more general published information.^39^ Volunteers need to assure themselves and their families of the capabilities of the sponsor organizations; a list of non-governmental organizations that meet certain criteria is maintained by the National Voluntary Organization Active in Disaster (NVOAD).^40^ Sponsors should have proven track records of effective humanitarian medical response missions in resource-poor countries, including their ability to maintain an adequate supply chain of necessary PPE.^21^ They must also provide a clear understanding of what the volunteer’s roles and responsibilities are likely to be in order to limit misunderstandings on-site. Volunteers can pre-register with sponsor organizations in order to obtain advanced verification of credentials and licensure, which can then facilitate rapid deployment.^41^

Potential volunteers should also avail themselves and their families of the many excellent no-cost training basic disaster preparedness programs so that they are better prepared to serve as a disaster volunteer. Information on these programs is noted in Table 2. Training programs that address cultural competency and cross-cultural job performance may also of value to volunteers. With information provided by the sponsor on the prevailing cultural norms of the host country, volunteers can become more competent in this regard. A good review of this is provided in a 2009 RAND publication.^42^

**Table 2:** Table 2. Recommended Resources

Resources	Website
Basic Disaster Training	
FEMA ICS Training - ICS-100, ICS-200	https://training.fema.gov/nims/
FEMA CDP	https://cdp.dhs.gov/
CERT	https://www.fema.gov/community-emergency-response-teams
Medical Reserve Corps	https://www.ready.gov/medical-reserve-corps
Médecins Sans Frontières	http://www.msf.org/
WHO	http://www.who.int/en/
Specialized Training	
CDC Ebola Toolkit	http://www.cdc.gov/vhf/ebola/hcp/safety-training-course/training-toolkit.html
CDC Continuing education - ETUs training	http://www.cdc.gov/vhf/ebola/hcp/safety-training-course/continuing-education.html
OSHA - PPE	https://www.osha.gov/Publications/osha3151.html
OSHA - PPE Assessment	https://www.osha.gov/dte/library/ppe_assessment/ppe_assessment.html
Patient Care & Ethics	
AHRQ - Altered standards of care in mass casualty events	http://archive.ahrq.gov/research/altstand/altstand.pdf
CA Hospital Emergency Preparedness - Crisis Care	http://www.calhospitalprepare.org/crisis-care
PHG Foundation - Principles of Bioethics	http://www.phgfoundation.org/tutorials/moral.theories/6.html
Red Cross - Ethics in Disaster Response	http://www.ifrc.org/en/what-we-do/disaster-management/responding/ethics-in- disaster-response/
Psychosocial Toolkit	
Reliefweb/Red Cross - Psychosocial support toolkit	http://reliefweb.int/report/world/caring-volunteers-psychosocial-support-toolkit
Johns Hopkins - Public Health Guide for Emergency	http://www.jhsph.edu/research/centers-and-institutes/center-for-refugee-and-disaster-response/publications_tools/publications/_CRDR_ICRC_Public_Health_Guide_Bo ok/Public_Health_Guide_for_Emergencies
Volunteer 101	
Harvard Medicine - Advice for deploying to a medical disaster	http://hms.harvard.edu/news/harvard-medicine/advice-deploying-medical-disaster
Medical Teams - deployment	http://www.medicalteams.org/take-action/volunteer/disaster-response-volunteering
PHE - Disaster Medical Assistance Team	http://www.phe.gov/preparedness/responders/ndms/teams/pages/dmat.aspx
Acronym list: FEMA: Federal Emergency Management Agency; ICS: Incident command system; WHO: World Health Organization; CDC: Center for Disease Control and Prevention; ETU: Ebola Treatment Unit; OSHA: Occupational safety and health administration; PPE: Personal Protective Equipment; PHE: Public Health Emergency.	

The responsibility of the sponsor should not end when the volunteer’s tour of duty ends. If volunteers have to be quarantined upon their return home, the sponsor should ensure they have policies and protocols in place to support the volunteer during quarantine. The sponsor should also ensure that the volunteers have mental health care resources available at all stages of deployment. A good resource for additional information on the maintenance of psychological well-being of volunteers is provided by the Substance Abuse and Mental Health Services Administration (SAMSHA).^43^ They urge disaster responders to take advantage of their extensive resource list to help them maintain their psychological well-being before, during and after a disaster deployment. Participation in support groups comprised of volunteers may also be advantageous.

Mental health aspects of deployment are of particular concern. We noted a striking similarity between post-deployment feelings reported in this study with those reported by returning war veterans. Recent scholarship on a relatively new phenomenon referred to as ‘moral injury’ may have some relevance here. Moral injury is believed to result when military personnel are confronted with ethical and moral challenges that transgress deeply held beliefs and morals.^44^^,^ 45 In our study, participants frequently witnessed suffering, undignified death, and limited ability to provide quality of care—and these exposures may have resulted in moral injury as the symptoms commonly associated with moral injury (shame, guilt, remorse, and demoralization)^45^ were frequently reported by our study participants. While moral injury is different from post-deployment mental health problems such as Post-Traumatic Stress Disorder (PTSD), co-morbidities with this and other mental health disorders are not unusual.^45^ Since the short and long term mental health impact of hazardous medical mission deployments has not been well characterized in volunteers, additional studies on this are warranted.

Despite the potential study limitations, which include small sample and self-report, selection, recall and social desirability response biases, important, in-depth information was obtained that can inform our understanding of the potential adverse impacts of high risk medical deployments. Additional studies are warranted to develop and evaluate best practices preparedness programs for high risk medical missions. There is also a need to assess pre-deployment counseling on health and mental health outcomes in medical volunteers.

## CONCLUSIONS

The urge to do something to help in an international medical crisis is understandable and admirable, but the adverse impacts reported by participants here – at every stage of deployment, suggest that preparedness for these missions needs improvement and at the very least, high risk missions should be limited to more seasoned and well trained (for conditions in the field) personnel. While volunteering for a medical mission during a health crisis can be very rewarding, both professionally and personally, it can also be very disruptive and impactful. All volunteers for high risk missions must be made fully aware of the risks they are going to face and the potential consequences, which may include illness, quarantine, and even death. The challenges of delivering care under extremely constrained conditions may be overwhelming for some people, and it is imperative that volunteers have realistic expectations of conditions in the field as well as a good understanding of how this deployment may affect their lives – before, during and after deployment.

Organizations sponsoring medical missions have a great responsibility to protect the safety and security of their volunteers by providing them with adequate training and ensuring that the medical missions are well managed. They also must ensure that their volunteers have appropriate counseling and mental health care resources available at every stage of their deployment. Support for a national (US) program that can provide oversight of high risk medical missions is advisable, especially since it is inevitable that there will be future outbreaks. Because the health and safety of volunteers for these types of missions may be at risk, preparedness of all key stakeholders is essential. The heroism of Ebola mission volunteers was appropriately honored by the 2014 Times ‘Person of the Year Award,’ and we can further honor their bravery and sacrifice by ensuring that future volunteers to high risk missions will be as well prepared as possible. Such efforts will serve to protect the volunteer as well as the international community, including the US.

## Corresponding Author

*Correspondence concerning this article should be addressed to Robyn R. M. Gershon, MHS, DrPH, Institute for Health Policy Studies, University of California, San Francisco. Tel: (415) 476-1890. Email: robyn.gershon@ucsf.edu.

## Competing Interests

The authors declare that they have no competing interests.

## Data Availability Statement

All relevant data are within the paper and its Supporting Information files.

## Appendix

**Appendix 1:** Appendix 1. Summary of experiences, key findings and recommended strategies for effective health care volunteerism on medical missions

Pre-deployment Phase			
Experiences	Key findings	Comments	Strategy recommendations
Motivation to volunteer	- Sense of ethical obligation - Social justice - Health equity - Prior humanitarian experience	During large scale humanitarian crisis events there may be shortages of volunteer workers.	Support concepts of ethical obligation, health equity, social justice and community service into all higher education health care programs in the US.
Fear for safety of self and significant others sense of isolation	- Developing fears and concerns of volunteers were internalized, not shared with family or others. - Some families were previously unaware of volunteer intentions	The actions of a volunteer impacts not only themselves, but also their significant others.	Build into large scale volunteer missions psycho- social support for volunteers and their families. Begin such support before deployment, stress importance of family inclusion in decisions and planning and implement a mechanism for frequent check-ins with their families.
Variability of training for deployment	- Volunteer preparedness varied from comprehensive training in Anniston GA, to simply being handed a brochure about EVD. - Some training did not include cultural education about the host country.	All relief agencies have a moral obligation to provide adequate preparation of volunteers before deployment, and the host country should demand it. Such training can provide bio-psychosocial protection for the volunteer, and also serve to reduce the likelihood that the volunteer will return to their home country with an infectious disease that might easily spread.	Provide training at two levels: pre-deployment and onsite: 1) Pre-deployment training must include content that address response, safety, self- care and local culture; 2) Ongoing training should continue at the deployment site in response to the local situation or recognized problems. In essence, there should be ongoing quality assurance of the relief effort with rapid response and training to address needed corrections. This will provide an ethical, efficient relief effort.
Peri-deployment			
Experiences	Key findings	Comments	Strategy recommendations
Initial volunteer culture shock	- Volunteers were not prepared for the severity of the austere conditions and limited resources.	Most Americans cannot imagine the conditions of severely under-developed countries, and pre-deployment preparations should focus not only on what they will be doing, but also on anticipated conditions in the field. There is a mismatch between what is envisioned and what is experienced upon arrival to the host country.	Pre-deployment training should include preparation for host country conditions. Simulation centers such as the CDC training center in Anniston GA or US military pre- deployment simulation centers can be used to simulate what the environment will actually be like, including sights, sounds, smells and the austere environment. The US government should support such simulation centers and make them available for use by NGOs when Americans are being deployed as volunteers.
Lack of organization and role clarity	- Responder roles were not clear. - Use of personnel was not efficient.	The international community has learned from many prior large scale response efforts that use of some version of the incident command system (ICS) serves to bring order and efficiency to disaster response efforts.	The host country and lead NGO agency should agree beforehand on a version of incident command that will be used to support the response. All other response agencies and volunteers should be trained on this incident response structure. All US volunteers should have knowledge of the Incident Command System used in the US. Such knowledge will support a more rapid understanding of the command system in use for the volunteer engagement. FEMA ICS training can be taken online and is free of charge.
Lack of resources	- The lack of resources in all categories (personal, space, supplies, equipment, policies, utilities, water) was a shock and disturbing. - Witnessing death was difficult and disturbing.	The mismatch between the required vs. lack of resources is the definition of a ‘disaster.’ Volunteers need to be prepared for not only the response to a specific event, but also require knowledge in basic disaster preparedness and the essentials of public health. Such knowledge will better equip them with skills needed to cope with a severe lack of resources and support innovation and adaptation as needed.	Pre-deployment training should include basic disaster preparedness and essentials of public health. Many courses are available, usually at no cost. Participation in this type of training will further serve to build capacity within the US for response to disasters in our own country (which are increasing in numbers and severity) as well as abroad.
Fears for safety of self	- Fears of contracting Ebola and psychological stress and fears related to personal security were present.	The fears of the volunteers were justified. EVD is a highly contagious disease and the lack of resources and apparent sponsor agency disorganization represented a real threat to the volunteers. This is where the host country, relief agency NGOs, volunteer home country and the volunteers themselves all have an obligation to do their part to provide the highest level of protection possible. Not all risk can be eliminated, but a well-trained volunteer workforce that is embedded in a relief effort that is well organized and coordinated can reduce such risk. Large scale disasters can precipitate or aggravate societal violence. Host countries have an obligation to put measures in place to protect relief effort of volunteers.	A high degree of host country and NGO coordination, cooperation and communication, as well as use of an ICS safety officer (whose job it is to assure responder safety) goes a long way to assure an environment that supports worker safety. Adequately prepared volunteers can mitigate many of the threats to volunteer bio- psycho-social safety. People who feel that they will be protected from harm during disaster response are more likely to serve as volunteers. Efforts to assure volunteer safety serve to increase access to larger numbers of qualified volunteers. Volunteers must be provided with personal safety training which includes relative risks, curfew hours, the need to travel in groups, local customs regarding gender behavior, manners, dress and communication. Such efforts can reduce the risk for volunteers.
PPE & Infection control protocols	- The required PPE is uncomfortable and cumbersome. - Infection control protocols were in place and the volunteers tried to adhere to infection control protocol.	The PPE required for response to Ebola and other highly contagious diseases is uncomfortable and cumbersome to use- but essential. Infection control protocols are necessary and support safety for the workers as well as limiting spread of the disease.	US efforts should continue to support and expand research and development efforts for new methods and materials for healthcare PPE that is effective, but more comfortable and less cumbersome. Improvements in PPE technology will improve US capability to response to bioevents in the US as well as abroad. Volunteers should be thoroughly trained in infection control protocols and use of PPE before deployment, and retrained upon arrival to the bioevent response site, and assessed for compliance on an ongoing basis with re-training as necessary.
Altered standards of care and ethical conflict	- Working with altered standards of care was difficult and contributed to ethical conflict.	Altered standards of care are inevitable during most large scale disasters such as the Ebola epidemic in Africa. Volunteers must be prepared to work with altered standards of care. Such understanding may serve to mitigate some amount of ethical conflict. However, basic emergency preparedness training can better prepare volunteers to understand the need for fluidity in terms of standards of care. Volunteers should be prepared to do the best that one can with the availability of resources at hand.	Pre-deployment training regarding altered standards of care, doing the most good for the most number of people with the resources on hand and the need for adjusting standards of care as more resources become available should be a routine part of volunteer training. Volunteers should be provided with updates regarding the resource situation and expected standards of care on a daily basis by the on-site Operations Commander. The Incident Command Structure should be adopted at all high risk deployment facilities.
Fatigue	- Fatigue was frequent, and rest periods were sometimes ignored.	Fatigue is not healthy for anyone and has been shown to reduce healthcare worker productivity and increase the risk of injury/exposure. In a dangerous EVD environment, extreme fatigue is likely to increase risk for breaches in infection control protocol. This then poses a heightened risk for other workers and patients.	Ordered rest periods should be mandatory and monitored for all workers. This should be enforced by a designated on-site Safety Officer.
Post-deployment			
Experiences	Key findings	Comments	Strategy recommendations
Volunteer quarantine	- There was significant stress related to quarantine upon return home to the US. - Some returnees and families were stigmatized. - There was a sense of lack of appreciation for volunteers’ efforts.	Being placed in quarantine upon return to the US and lack of appreciation for their efforts came as a surprise for which some of the volunteers were not prepared. During an event such as Ebola epidemic, where unknowns may exist about the true risk for transmission, poses a significant challenge for public health officials. Basic disaster preparedness training would better prepare volunteers to understand that low risk but very high impact events (such as transmission of Ebola by a volunteer to citizens) create public health concerns that can require drastic response that includes erring on the side of caution. This dilemma illustrates how volunteering impacts not only the volunteer, but also their family and community. This is a controversial issue that will persist for events such as EVD.	During large scale events such as the Ebola epidemic that US citizens are likely to volunteer for, the associated risks to volunteers should be assessed and honestly reported by the appropriate US governmental agencies to the populace. Plans for management (actual or potential) of volunteers who return in order to protect the US population should be posted on the CDC website. Standards of management of returning volunteers who are placed in quarantine should be made by the CDC, so that those who are quarantined are treated humanely to reduce their sense of isolation. Pre-deployment basic disaster preparedness and event-specific preparedness training that includes potential post-deployment quarantine will serve to better inform potential volunteers. This will provide them with the knowledge needed to make choices regarding the decision to volunteer and help them plan for potential post-deployment consequences for themselves and their family. Open, honest dialogue about these issues will help avoid adverse consequences. Volunteers need to have a clear understanding of the commitment they are making. Widespread community education is required so that the US citizenry understands what safety controls are in place to help keep them safe.
Emotional impact of volunteering	- Some reported grief, depression, anxiety upon leaving. - Others expressed self-doubt and re-entry stress.	Serving and being witness to large scale disaster events can have lasting impact on volunteers. This impact can be both negative and positive. More resources are needed on the issue of emotional safety of volunteers to events such as the Ebola epidemic. It is thought that pre- deployment preparation can provide some element of a protective factor, in that the volunteer will better anticipate the shocking conditions and thus be better prepared. Perhaps comprehensive pre-deployment preparation and training is more likely to provide the volunteer with a better sense of control by reducing uncertainly. All of these factors and the efficacy of volunteer preparation need to be studied.NGOs have an obligation to assure the safety of the volunteers to the extent possible. This includes support for the volunteers’ post-deployment so that their re-entry can be supported.	The US should fund research that examines factors related to volunteer emotional protection and safety and the effect of various interventions to protect volunteers from psychosocial and emotional harm.
Acronym list: EVD: Ebola virus disease; ICS: Incident command system; NGO: Non-governmental organization; FEMA: Federal Emergency Management Agency; PPE: Personal protective equipment			
